# American Academy of Dental Science

**Published:** 1893-07

**Authors:** 

**Affiliations:** American Academy of Dental Science


### AMERICAN ACADEMY OF DENTAL SCIENCE.

The regular monthly meeting of the American Academy of Dental Science was held in the Boston Medical Library Association rooms, February 1, 1893, at 7.30 P.M. President Brackett in the chair.

*President Brackett.*—The subject of our meeting is "Crown- and Bridge-Work," and Dr. Moffatt will open the discussion.

### DISCUSSION.

*Dr. Moffatt.*—It is not my purpose to present an elaborate treatise upon the subject of crown- and bridge-work.

The "American System of Dentistry" devotes nearly two hundred pages to this subject, presenting such a variety of methods that we find ourselves in a labyrinthian maze, and feel as if we were looking at a railroad map of the United States, with all the stations in capital letters.

Professor Litch commences his article by saying that for the systematic study of constructive details in tooth-crown and bridge-work, the model of an upper palatine arch, with two strongly-planted cuspid roots and two twelfth-year molar teeth or roots, may be taken as "a typical case." That may do for a study, but, in my opinion, it is far from being a typical case. It reminds me of the cut that was so profusely circulated in the New York daily papers some years ago. The public were invited to bring their mouths, with four such snags in them, and have a piece of permanent bridge-work inserted that would last forever.

Now, such ready-made cases may present themselves, and those who advertise in such a general way may be able to collect numerous cases of that type. But, in my experience, such typical cases are very rare in general practice. Each case that presents itself is

*sui generis*, and must be studied by itself, and talent and skill exercised in its treatment.

No doubt Dr. Richmond has had as much experience as any or all of us, and it is very interesting and instructive to see him work in his own peculiar way. He has a great many little ideas that cannot be readily explained, and can only be understood by seeing him work. He has almost entirely abandoned the use of permanent bridge-work for large cases, using it only in small and limited areas, and, in my estimation, this is the best practice.

Bridge-work, as you all know, is not entirely the child of this generation. I was instructed in making bridges when I was a student, over thirty years ago. Bridge-work was done in those times; but, of course, modern methods have greatly improved the operation and extended its employment to a greater number of cases.

*Dr. Smith.*—At the request of the chairman of the Executive Committee I present models of a case of crown- and bridge-work. Many of you, I think, have seen the case before, but you have probably forgotten it, and it may be new to you now.

This is a case of crown- and bridge-work, and also, in connection with it, contour-work with cohesive gold. This model, marked "forty-two A," represents the case as it came to me. The result of loss of molars and their wearing down, and also the wearing down of palatal surfaces of the incisors and cuspids, is evident. One cuspid was a pulpless tooth; the other was alive. The pulp of the latter was destroyed to facilitate the better adaptation of the bridge-work, and after the bridge was set, gold fillings were put into the cuspid teeth, and they were built up in the same way as the centrals.

This case was done eleven years ago, and I have never had any trouble with it, nor has the patient. It is the only extensive bridge case that I have done. I have always questioned the advisability of permanent bridge-work, but this case appeared to me at the time to be particularly adapted to it, and the operation was performed.

*President Brackett.*—It seems to the Chair always gratifying to hear with reference to any structures of this kind that have borne the test of time. That is one of several good points that Dr. Smith has brought out in his statement in regard to this case.

*Dr. Banfield.*—Recently I replaced a permanent bridge with a removable one. It was composed of two bicuspids and a molar. Caps were made for the molar and bicuspid roots, and cylinders with the bridge attached made to slip over the caps. If an accident should happen, I can slip off the bridge and repair at my leisure.

*President Brackett.*—The Chair would like to ask if this bridge-work is supposed to be removed by the patient.

*Dr. Banfield.*—No, sir; the cylinders fitted so closely I was obliged to file them, for fear they could not be removed after being adjusted to the mouth. Even now it will take considerable force to remove the bridge.

*Dr. Meriam.*—I have had almost no experience in this bridge-work, and, I am very thankful to say, quite a limited experience in crowning. But from what I have seen it has appeared to me that we depended too much on soft preparations of gold for the support of bridge-work, and that the tendency of gold to spread in wear is a very important cause of the failure of such work. If some of the bridge-work was started with a little stiffer form of gold, there would of course be more trouble in fitting, but it would undoubtedly give better wear. The amount that a piece of twenty-two-carat gold will yield is considerable.

*Dr. Moffatt.*—I quite agree with Dr. Meriam that twenty-two-carat gold is too soft to use in many cases. I had a case recently which gave me considerable experience in that direction. The soft gold of which seamless bands are made had to be abandoned, and a stiff, heavy platinum clasp-plate used in its place. In a great many narrow bridges, where you want a very little plate, but still a very stiff appliance, a large flow of solder over a platinum and iridium wire, finished round or half-round, makes a very stiff and rigid plate.

*Dr. Cooke.*—I should like to ask to what extent the members have used crowns that are already prepared. The idea one gets from the cuts in the magazines is that all you have to do with ready-made crowns is to trim them up a little and press them into place.

*Dr. Meriam.*—I had in mind a crown put on by a neighbor of mine when I spoke about using soft gold. The band was easily spread, and was pressed into place so as fit the festoon of the gum beautifully; but it was soon found that the crown that was so easy for the dentist to spread and put on proved equally easy for the patient to spread and put off.

*Dr. Allen.*—I have attempted in two or three instances to use these ready-made crowns, but in each instance I have failed to secure satisfactory results, and have abandoned the attempt and finished the operation by making crowns in the usual way. I feel that I can work with better facility when making the crowns myself than by using ready-made ones.

*Dr. Banfield.*—Does Dr. Allen think that if these ready-made crowns were composed of eighteen- or twenty-carat gold, and of number thirty gauge, they would be of service?

*Dr. Allen.*—My trouble has not been with the yielding of the crown, but in adapting the band to the root and in getting the proper articulation. If I get the band over the root, I am not always sure of getting the crown in proper occlusion.

*Dr. Grant.*—Quite a little while ago I read an advertisement in one of the dental journals, in which some man—I think it was Dr. Land—claimed that he could attach amalgam or gold firmly to porcelain. I thought then that if he did it he must do it in some such way as the china painters put on gold in decorating. To see if it could be done, I made a solution of gold in aqua regia and precipitated it, washed out the precipitate, and after drying it, made a flux with that and nitrate of bismuth, using plenty of gold. I then made several crowns and set them on molar roots with amalgam. The amalgam made an absolute union with the heavily-gilded surface. You can get enough gold on a tooth by this method so that it will hold amalgam firmly. There is no question but what it is an absolutely perfect joint. I intended afterwards to try borax as a flux, but I never did it.

*Dr. Meriam.*—I think the receipt-books all give an amalgam that will adhere to glass without difficulty, and I have thought that one of these days we would find a metal that would adhere to porcelain in the same way.

*Dr. Cooke.*—I would like to ask Dr. Grant if he relied on the union of the amalgam and the piece of porcelain to hold the crown.

*Dr. Grant.*—Oh, no. The object is to get a joint there; but by getting such a complete union between the amalgam and gold the tooth is made additionally strong. You cannot do it with decorators' gold, because there is not enough gold there; you have to make the flux yourself.

*Dr. Cooke.*—I am not quite satisfied with this discussion. I wanted to learn something about crown- and bridge-work, and I should like to find out what the members think is the best crown to use; and I wish, Mr. President, you would commence and ask each one separately what method is preferred.

*Dr. Brackett.*—Suppose we begin with you. What kind do you prefer?

*Dr. Cooke.*—I think a banded crown is the best one to use. Have the band go around the root and under the gum, and the pin inside set with cement. I prefer that to the crown that is simply held by the dowel.

*Dr. Banfield.*—If I were asked what was the best crown to use, I should be obliged to say, "It depends upon the case." At the present time I know of no one crown that would be well to use in all cases. In one I now have in mind, where the front teeth are short and very far apart, with sound roots, I cannot conceive of a kind of crown any more satisfactory than the all porcelain. A crown with any gold would look badly. I am soon to put on some crowns where the roots are weak and badly decayed at the labial surface, with recession of the gum. In such a case I would use a gold band to strengthen the roots.

The trouble I find with the Logan crown is that it is difficult to find one whose neck fits the surface of the roots; it is either too large or too small.

*Dr. Preston.*—I have nothing to say about crowns, Mr. President, but I brought a piece of bridge-work to show you, which I made July 29, 1839, and which was in use for nine years. I do not recollect that I ever did anything to the teeth until they were taken out. I made this without knowing anything about crowns, and it was intended to be permanently fixed in its place, being set in the roots with wood, and having gold pins in the two front roots. I put in a number of cases about the time at which this was done.

*Dr. Moffatt.*—Mr. President, I think that Dr. Preston more than bears me out in my statement that bridge-work is not a child of the present generation. Here is something that goes back over half a century.

*Dr. Barker.*—I believe Dr. Cooke's object was to bring out the preference of the different members for some special crown.

Probably practitioners will usually prefer that method which is to them the easiest. For a long time I used the Richmond crown pure and simple,—the band and the porcelain crown backed up and soldered to the band. I made and mounted numbers of them until Dr. Stowell, of Pittsfield, showed me in Montreal, a number of years ago, a method of his own. Since then I have mounted crowns according to the Stowell method. A description of his method can be found in the "American System of Dentistry." It is a simple banded crown, made by cutting off close to the porcelain the pin of a Logan crown, flowing pure gold around the pin, and soldering that bit of pure gold to a band which you have already fitted to the root. It is for me a simpler method and more certain than the

Richmond, and is preferable, particularly in the case of front teeth, as nothing is seen excepting the porcelain.

One of the principal objections I find to the Richmond crown is that you cannot rely upon your colors, and in one or two cases I found it very annoying. With the Stowell method you do not have this difficulty.

For a simple pin crown I use the Logan entirely. I consider it strong and simple and satisfactory in all points, with the exception which Dr. Banfield spoke of,—the fact that the neck is often too narrow.

*Dr. Bradley.*—My experience in crowning, Mr. President, has been somewhat limited. I have used the Richmond crown for a banded crown, mounting it with gutta-percha; but I use more of the Logan than I do of the Richmond, and I should say with considerable success. I have had a few roots split where a Logan crown was used, and also where the Richmond crown was used, though more rarely in the case of the latter. I use Dr. Evans's gold caps to a certain extent, and with what seems to me good success. There is one case which I would like to speak of. The patient was an officer in the army, who had nothing left of the right inferior first molar but two roots, and he asked me if I could not do something with them. The crown was entirely gone, and the roots were not even standing straight,—one of them laid over on the side. He said that food continually getting in these roots bothered him, but he did not want to have them taken out if it were possible to make any use of them. I took an impression of the roots in modelling composition and sent to Dr. Evans for two of his crowns, and after fitting them on, invested them in asbestos and plaster and soldered the two caps together and fitted them. The roots were not quite firm when I put the crown on, but they are a little firmer now. The gentleman says it is the best tooth he has in his mouth, and he gets a great deal of satisfaction from it. So far, I have been well satisfied with Dr. Evans's caps, and know of no case yet where there has been any difficulty in my use of them.

*Dr. Grant.*—I don't believe much in banded crowns. I have never had very good success with them. I don't think it is really necessary once in twenty cases to band a root. Of course I have seen a great many crowns that have been in use a number of years. I saw one not long ago that was put in by Dr. Moffatt over twenty years ago, before much banding was done. It was in the form of an ordinary pivot tooth, except the socket in the porcelain was of platinum, and there was a gold wire running through the wood pivot pin, but the joint was just as good when I saw it as it was when it was first made.

A most important feature in any crown is to be able to remove it. Of course every one knows that there is no porcelain crown that will not break, and when one does break, it is a tremendous job to take the pin out. It takes more time to get it out of the root than it does to put it on, if you want to save the root. A method of crowning that I sometimes employ is to take a piece of gold tubing, which is cut to a length equal to the depth of the root-canal. Drive a square cutting tool through the gold tubing, and make a perfectly plain-sided socket similar to that of a watch-key. This tube can be set in any way you prefer, and a square pin is made to fit it. The tooth is soldered to this pin, and when the tooth, thus mounted, is placed in the socket, I have never known one of them to come out except by a straight pull. Of course they are liable to be broken, but new teeth can easily be put on.

Another plan is to bake a square or three-cornered platinum pin in the old-fashioned pivot tooth. The pin can be secured firmly in the crown by a flux of powdered flint glass and borax. Continuous gum body will answer, but the glass requires less heat.

In this way the relation of pin and crown are easily adjusted, while the result, in point of strength, is quite equal to that obtained by the Logan crown.

*Dr. Meriam.*—I would first, Mr. President, advocate the use of nitrate of silver in the treatment not only of the ends of the roots, but to a certain extent of root-canals, after preparing them for the pins.

Regarding a banded crown, if the joint is to be conspicuous I might not use one. For back teeth I should use the gold-band crown for men, and in all cases where the crown was to be subjected to severe use. For bicuspids I would prefer the English tube tooth. The colors are so good that I submit patiently to the annoyance of grinding them to a fit, and they are big enough at the necks of the teeth to cover the roots. The Howland crown seems to me a very valuable crown where there is room for it. It requires a certain depth of the crown to hold the pin, and that may be an objection; and another is that it has had no one to push it, but has depended entirely on its merits to bring it to prominent notice. It is only just now receiving the attention that it should.

For back teeth I prefer a crown that I can remove, and also prefer to use gutta-percha in the roots, except where the canal is large; then I use cement.

*Dr. Stanton.*—The subject of crowns has been so thoroughly covered that it seems almost impossible for one to add anything to it. My extensive experience, to which Dr. Smith has referred, has been more in the way of repairing other people's work than in doing new work. When I first began practice, a large number of patients who had had crown- and bridge-work done for them fell into my hands. I have, consequently, formed some very decided opinions with regard to crowns, simply from the results of the experience of others.

From a personal stand-point I think the only fit crown to use is one with a band on it. I have seen so many fractured roots and so much decomposition of the end of the root from loose joints that I cannot but feel that the slightest play between the end of the crown and the root is fatal to the success of the operation. It does not matter how slight the movement may be, you are bound to have decomposition where it exists, and, strange as it may seem, it will follow the pin up into the root.

The recession of the gum and consequent showing of the band have been spoken of. I think a Richmond crown can be put on incisors without the band showing at all.

I do not think that the presence of the gold or platinum causes any recession whatever. I have seen banded teeth which have been in use for five to fifteen years, and, so far as I have been able to discover, the gums do not show the slightest indication of receding. If you put an all-porcelain crown on an incisor, you are dependent entirely on the pin for the strength of the tooth, and if there is heavy pressure brought to bear upon it, then either the porcelain crown will give way or the root will split. These results, of course, will reflect upon your skill and judgment. To my mind the lesser evil would be to put a band around such a root, even though it did show somewhat. But I have found, if a tooth is properly ground with a very thin corundum-disk, that you can carry the thin edge of the band under the gum in such a way that it does not show.

Supposing, however, that the band does show, it is infinitely more to your credit to use it, and have as a result a tooth that will last until the patient is obliged to lose his teeth from natural causes, than to endeavor to perform an æsthetic operation which will look well, but which is unreliable.

Cases of bridge-work come to grief from one of two causes: either decay will have progressed sufficiently to loosen the caps,—a process which is fatal to the whole bridge,—or there will be so much pressure brought to bear upon the supports that a tooth is pulled out. The latter condition may be found in removable bridges.

*Dr. Smith.*—I would like to ask Dr. Stanton whether, after considering the evils of both permanent and removable bridge-work, he believes in bridge-work of any kind.

*Dr. Stanton.*—That is a very difficult question to answer. Many cases of bridge-work are very successful. I know of an instance where a person has worn a full upper set and two partial bridges on the lower jaw for fifteen years, and they seem to be in as good condition to-day as when I first saw them. You will often meet with bridges supporting one-third to two-thirds of an upper or lower set which have been used for years and are still perfectly solid. On the other hand, one out of the next half-dozen that you see will be loose from a cause which you will never learn, because it is impossible to obtain the history.

Bridge-work was very popular at one time, from the prevalent idea that loose teeth would be tightened by its use. There never was a greater fallacy in the world. One of the essentials for a successful bridge is to have firm foundations. Even then it is impossible to say that it will surely be a success. I never guarantee that a piece of bridge-work will last. Where bridge-work can be put in and the structure made light, a removable bridge is preferable to a permanent one. I have found it interesting to note how much some people will have bridges repaired before they will give them up; they seem to have an affection for them, and will pay enough in the way of repair to get a new appliance or an artificial plate.

*Dr. Smith.*—Dr. Banfield referred to a crown which he said I was very enthusiastic over. It is simply an idea of putting a hole in a crown, which I got from Dr. Perry, of New York. In the old-fashioned incisor pivot tooth the hole took such an angle that the tooth was made very weak. In case the tooth was ground for an occlusion, it became weaker still. Dr. Perry had some crowns made with the pivot hole pointing straight towards the cutting edge, and the result is a much stronger tooth.

No more artistic work in prosthetic dentistry can be done than to crown a root in the front of the mouth so that it will appear entirely natural. I had a case where I destroyed the pulps in the six front teeth and put on crowns. The method I used was to cut the labial portion of the root down under the margin of the gum and fit accurately a Perry crown. A platinum and iridium pin was set in the root-canal with gutta percha, and then the crown set with cement. In doing this work crowns had to be carved for the case and many had to be carved before satisfactory ones were obtained. While waiting for the carved teeth, ordinary teeth with very short holes were used.

In order to remove crowns set in gutta-percha, Dr. Payne, my associate, suggested the following plan: A small bottle was fitted with a cork, and through the cork a tube was passed. Through this tube a few threads of wicking were drawn, and thus an alcohol lamp was made which gave a miniature flame. This flame could be carried to the tooth, and sufficient heat applied so that it could be easily removed.

*President Brackett.*—Dr. M. W. Foster, of Baltimore, years ago, gave me an instrument which, in its use, bears a resemblance to the miniature alcohol lamp that has been spoken of. It was made presumably of soft steel, with a handle of wood. The steel end had a deep V-shaped groove, and it was intended to be heated and slipped over the crowns to facilitate their setting in gutta-percha and their removal.

Another means of applying heat is by a continuous blast of hot air, produced by a syringe. With this a tooth and its setting may be made very hot.

*Dr. Meriam.*—Dr. Baycock sent me a little lamp of the same sort as Dr. Smith describes, except that it was made from a medicine-dropper instead of a bottle. It can be used as a small lamp for heating or as a flash lamp for cavities.

*President Brackett.*—It is hard to draw the line between this discussion and the demonstration of the use of a new furnace for porcelain fillings and crowns, by Dr. Bartlett. If Dr. Bartlett is ready, we should be glad to have him explain it to us.

Dr. S. R. Bartlett exhibited a small gas furnace, made by the Detroit Dental Manufacturing Company, designed for baking porcelain fillings and crowns. The muffle, which is made of platinum, is just large enough for one or two teeth, and has a platinum tray for holding the piece to be baked. Dr. Bartlett gave a demonstration of the use of this furnace by baking an incisor crown in just two minutes. The tooth used was an ordinary plain rubber tooth with iridio-platinum dowel held in position between the pins of the tooth, the band for the root being made of platinum with an investment of silex and plaster which surrounded the upper portion of the dowel and inner space of the band. The tooth thus invested was formed to the proper contour by Allan's continuous gum body, which covered the labial part of the band and was made continuous with the neck of the tooth. The crown was then baked; the time required for heating, baking, and cooling being about ten minutes. By this process of making crowns—which Dr. Bartlett does not claim as original—the objection to having the metal band show when the gum recedes is obviated.

William H. Potter, D.M.D.,
*Editor American Academy of Dental Science.*

